# Patient satisfaction with healthcare provided by family doctors: primary dimensions and an attempt at typology

**DOI:** 10.1186/1472-6963-9-63

**Published:** 2009-04-16

**Authors:** Ludmila Marcinowicz, Slawomir Chlabicz, Ryszard Grebowski

**Affiliations:** 1 Department of Family Medicine and Community Nursing, Medical University of Bialystok, 15 054 Bialystok, Mieszka I 4 B, Poland

## Abstract

**Background:**

Patient satisfaction is a complex and difficult concept to measure, thus precluding the use of exclusively quantitative methods for its description. The purpose of this survey was firstly to identify particular healthcare dimensions that determine a patient's satisfaction or dissatisfaction; and secondly to attempt to typologise the patients' responses based on their evaluation of healthcare.

**Methods:**

Using a qualitative research design, thirty-six in-depth interviews with patients of family physicians were conducted: four patients from each of 9 family practices in different regions of Poland were interviewed. The main outcome measure was factors associated with patient satisfaction/dissatisfaction.

**Results:**

In their evaluations of their contacts with family doctors, the patients cited mostly issues concerning interpersonal relationships with the doctor. Nearly 40% of the statements referred to this aspect of healthcare, with nearly equal proportions of positive and negative comments. The second most frequent category of responses concerned contextual factors (21%) that related to conditions of medical service, with two-thirds of the evaluations being negative. Statements concerning the doctor's competencies (12.9%) and personal qualities (10.5%) were less common.

**Conclusion:**

To improve the quality of healthcare, family doctors should take special care to ensure the quality of their interactions with patients.

## Background

Patients have a legitimate and important role as evaluators of healthcare. Obtaining feedback from patients about the quality of primary healthcare is a powerful way to develop more patient-centred approaches to healthcare delivery [1].

Satisfaction of patients is an important and desired outcome measure of the quality of care [2].

A number of new tools have been developed to measure patient satisfaction and quality of care in general practice [3-5]. However, patients from different countries and varied healthcare systems all report high level of satisfaction with general practice [6,7], which makes the results difficult to use in quality improvement. Satisfaction is a complex and difficult concept, and use of exclusively quantitative methods for its measure is inadequate [8-10]. This especially applies to efforts aimed at determining the components of satisfaction as they actually are, and not those accepted a priori by researchers. The former can be identified solely with the aid of open questions posed to the user of the service and enquiring about his/her personal experiences and opinions, and recording and analysing the responses. Such an approach increases the chance of identifying the elemental qualities of patient satisfaction instead of creating artefacts.

Some researchers have stated that patient satisfaction surveys are particularly inadequate for exploring dissatisfaction with healthcare [11]. Mulcahy and Tritter [12] argued that dissatisfaction and satisfaction are distinct constructs.

The purpose of this study was firstly to analyse the possibility of characterising the patients' responses typologically, taking into account patient evaluations of medical care; and secondly, to identify the particular healthcare dimensions that determine a patient's satisfaction or dissatisfaction.

Understanding patient satisfaction with the healthcare provided by family doctors is, first and foremost, essential for improving the quality of medical care.

## Methods

### Recruitment and sample

We used a maximum variation sampling strategy [13]. Nine family practices were chosen from different Polish regions to include both urban (big and small cities) and rural environments. In each of the practices, interviews were conducted with 4 patients coming in turn for consultation with a family doctor. The interviewer (LM) reported to the family practice, introduced the aim of the study to the patients and asked whether they would be willing to participate in the study. If yes, an appointment was arranged with the subject. Four persons (3 women and 1 man) refused to participate in the study without specifying the reason.

### Data collection

Interviews (lasting from 25 minutes to 2 1/2 hours) were based on an interview guide (see appendix 1). With some exceptions, the interviews were conducted in respondents' own homes. With the patient's consent, each interview was tape-recorded and transcribed in full.

### Data analysis

To interpret the respondents' answers, a content analysis was used [14]. Two researchers (LM and RG) were involved in analysing the themes which emerged from the data. Two researchers (LM and RG) read the interview transcripts. All words and expressions that concerned satisfaction or dissatisfaction were underlined in the transcripts. All categories mentioned by the patients were coded as positive or negative. For each interview, a matrix was prepared on which the patient's statements were coded. The method of categorisation of the analysed elements is shown in Table 1.

**Table 1:** Categorisation of the analysed components – examples of patients' statements

Category	Examples of patients' statements
Assessment of personality features	"A very nice doctor" (+*ve*)
	"He is arrogant" (-*ve*)
Assessment of competences	"She is a good doctor, her diagnoses are correct" (+*ve*)
	"She often prescribes drugs which are ineffective" (-*ve)*
Assessment of doctor-patient interactions	"Doctor talks to me and explains much to me" (+*ve*)
	"She did not ask me exactly what hurts" (-*ve*)
Contextual factors	"Fantastic equipment" (+*ve*)
	"I come in, nobody's there, and yet I had to wait 15 minutes" (-*ve*)
General assessment	"Everything is always all right" (+*ve*)
	"It was awful" (-*ve*)

The list of statements (positive and negative) in the respective categories is presented (see Additional file 1).

Categorisation of the above-mentioned elements was conducted independently by two researchers (LM and RG). The agreement in the scoring was good (κ = 0.78). Where differences existed, consensus was negotiated with a psychologist. In all the interviews, both positive and negative statements were observed, though in different proportions. As an indicator of the positive or negative character of each interview, a quotient of the number of positive and negative statements was adopted: i.e., a score of 1 signified an equal number of both types of evaluations: <1 meant a predominance of negative assessments: and >1 indicated a predominance of positive evaluations.

Quantitative data were analysed using Statistica PL v 7.1 software. The chi-square test was used to compare the distribution of positive and negative statements (Table 2).

**Table 2:** Dimensions of evaluation – positive and negative statements

Dimensions of evaluation	Positive		Negative		Total	
	n	(%)	n	(%)	N	(%)
Doctor-patient interaction	255	(49.6)	259	(50.4)	514	(39.4)
Contextual factors	92	(33.5)	183	(66.5)	275	(21.0)
General assessment	155	(73.5)	56	(26.5)	211	(16.2)
Competences of the doctor	87	(51.8)	81	(48.2)	168	(12.9)
Personality traits	100	(73.5)	37	(26.5)	137	(10.5)

Total	689	(58.8)	616	(47.2)	1305	(100.0)

## Results

Thirty-six in-depth interviews were conducted. The sample consisted of 20 women and 16 men, aged 20 to 78 years. Twenty-four respondents resided in cities, 12 in villages.

### A typology of patient responses – fundamental typology

Three main categories of responses emerged as the primary level of categorisation: "ambivalent" – 5 people; "positive" – 17 people; and "negative" – 14 people. The two latter categories appear to be internally diversified (from 0.12 to 0.95 for the first category, and from 1.09 to 9.67 for the second), which might justify creation of 5 or more categories (for instance "rather positive" and "rather negative"); however, this seems unnecessary in the present case.

### Typology according to content of the evaluations

Another basis for categorisation stresses the patient's perspective in healthcare evaluation.

The analysis distinguished the main factors associated with satisfaction or dissatisfaction. These were: personal traits of the doctor, the doctor's competencies, doctor-patient interactions, contextual factors, and general assessments that cannot be specified.

Thus, the following categories can be distinguished:

- orientated towards evaluation of the doctor's personal qualities,

- orientated towards evaluation of doctor's competencies,

- orientated towards evaluation of the doctor-patient interaction,

- orientated towards evaluation of contextual factors, and

- orientated towards expressing general opinions.

#### Orientated towards personality evaluation

In this category the most predominant statements concerned personal features of family doctors and their manners, for instance: "I really like this doctor, because he is nice, pleasant and sensitive; my type of a doctor." (Interview 34)

#### Orientated towards competence evaluation

This group comprises patients who value a doctor's competence and effectiveness the most. One of the characteristic statements is: "I read things that are on the information boards. I read what specialisations doctors have, what degrees they have, where they work, their achievements, where they have gained their work experience. It seems to be really important." (Interview 19)

#### Orientated towards evaluation of the doctor-patient interaction

For this group of patients the most important factor influencing their evaluation was the doctor-patient interaction, for instance: "Sometimes the doctor does not say anything, he examines me, takes some notes, doesn't say anything, and it is me who has to ask questions." (Interview 12)

#### Orientated towards contextual factors

Whilst describing the quality of care provided by a family doctor, some of the participants focused on the context of the situation in which the healthcare was provided. One of the patients said: "I have reservations about the receptionists, because very often I cannot get a hold of them." (Interview 10)

#### Orientated towards expressing general opinions

Some of the patients' statements were very general, for instance: "Here, everything is the way it should be." (Interview 28); "It is really nice in here." (Interview 28)

### Dimensions and evaluation of healthcare provided by family doctors with regard to the number of positive and negative statements

One of the most commonly used categorisations stated by the study participants – often the sole or fundamental one – was the level of satisfaction with medical care. This is actually the domain of quantitative research. In the qualitative type of research, this kind of categorisation is also possible, though it does not concern the participants but rather their utterances.

The lengthy transcripts from 36 in-depth interviews contained 1,305 statements to which a positive or negative connotation could be attributed: 689 (52.8%) were positive and 616 (47.2%) were negative (Table 2). The difference in the distribution of positive and negative statements was statistically significant (chi-square = 102.02; p < 0.001). In their evaluations of their contacts with the family doctor, the patients mentioned mostly issues connected with interpersonal relations with the doctor. Almost 40% of the statements referred to this very aspect of healthcare, with nearly equal proportions of positive and negative comments. The second most frequently mentioned were contextual factors (21%) which related to conditions of medical service, with two-thirds of the evaluations being negative. Statements concerning the doctor's competencies (12.9%) and personal qualities (10.5%) were less common. As for competencies, there was only a slight excess of positive statements over negative ones, whereas for personal characteristics, positive comments far outnumbered negative ones (by nearly three to one). The relatively large number of general statements (16.2%) suggests a tendency to dislike having to evaluate specific healthcare dimensions. Therefore, the preponderance of positive assessments (nearly three fourths) seems understandable. The comments were also accompanied by expressions of emotion (18.3% of the statements), with negative (19.2%) statements being slightly more common than positive ones (17.5%).

## Discussion

In our research we implemented a qualitative approach, focusing on research into patients' experiences [15]. We attempted to typologise the patients' characterisation of "satisfaction" with the quality of healthcare and to identify those family doctor care dimensions that decide a patient's satisfaction or dissatisfaction. This is important in order to understand better the process of patient evaluation, because our knowledge about the complexity of such evaluation is still unsatisfactory. On the other hand, better understanding of the patients' perspective is a prerequisite to fulfil patients' expectations. The results of our study allowed us not only to define the taxonomy of satisfaction dimensions, but also its relative importance for patients. Doctor-patient interactions appear to be valued the most, being found in 40% of the patients' statements; however, they are equally the cause of satisfaction and dissatisfaction. Whilst interpreting the results, it is important to remember that each patient's evaluations and statements concerned their family doctor's service – a setting in which a personal relationship between patient and doctor is especially important and desired [16,17]. Similar results were obtained by other qualitative studies, which aimed to explore the perceptions of patients with chronic conditions about the nature and quality of their care in general practice. In those studies, three categories of priorities were established. The first category concerned the doctors, i.e. their competence, interpersonal skills, time spent with patients in consultation, and continuity of care. A second concerned involvement of patients in their own chronic care, i.e. recognition of their own knowledge about their condition. The third focused on the practice team, i.e. the friendliness of receptionists and the helpfulness of practice nurses in providing patients with information about their conditions [18]. On the other hand, researchers from Scandinavian countries determined the following dimensions: communication, emotions, outcome, barriers, and auxiliary staff [3].

There is lack of a consensus in the literature concerning factors that determine patient satisfaction. For instance, Sitzia and Wood [19] singled out the following components of satisfaction: accessibility, interpersonal aspects of care, technical aspects of care, and patient's education/information. Some investigations aimed solely at evaluating the doctor's behaviour. For this categorisation, Hall et al. [20] employed a very interesting taxonomy, the usefulness of which was confirmed by Jung et al. [21]. In both studies, two principal dimensions in evaluating a doctor's behaviour were distinguished: task performance and effective performance. The sub-classes of the first category included: information giving, questions, action, and medical/technical competence, whilst the second category comprised socioemotional behaviour and partnership building [21]. Other research has provided evidence that patients evaluate the doctor's behaviour using two measures: professional competence and the display of empathy [17].

In our own research, a major role – mainly as a cause of dissatisfaction – can be attributed to the contextual factors pertaining to the conditions under which doctor-patient contact takes place. These conditions are known to cause justified reservations and criticism.

The relative balance established between positive and negative evaluations in the present research is characteristic solely of qualitative studies. In quantitative studies (usually in the form of a questionnaire), negative evaluations are the exception, because the research designs do not promote their detection [9].

## Conclusion

Patient evaluation of the healthcare provided by his/her family doctor is a multidimensional concept, its main component being the doctor-patient interaction. To improve the quality of healthcare, family doctors should take special care to ensure the quality of their interactions with patients.

Negative opinions expressed by patients differed qualitatively from positive ones; negative evaluations were associated with contextual factors, whereas positive one concerned doctors' personality traits and general assessment. Therefore, negative and positive evaluations should be referred to as two different concepts to be measured in different ways.

## Competing interests

The authors declare that they have no competing interests.

## Authors' contributions

LM was involved in the original idea, design, analysis and interpretation of the data, and wrote the major part of the manuscript. SC contributed to writing and the critical revision of the manuscript and the interpretation of the data. RG contributed to the revision of the manuscript and analysis and interpretation of the data. All authors read and approved the final manuscript.

## Appendix

The interview guide

1. What are your experiences with the use of family doctor services?

2. What does it mean to you to be satisfied with the visit at the family doctor?

3. Is there anything you are especially satisfied with?

4. In what circumstances are you dissatisfied?

5. Have you experienced any particularly dissatisfying situations?

What was your last visit at the family doctor's like?

## Pre-publication history

The pre-publication history for this paper can be accessed here:



## Supplementary Material

Additional file 1 **List of positive and negative statements in the respective categories**. The list of positive and negative statements in the respective categories is presented. Click here for file
