# Supplementary material for: Patient satisfaction with healthcare provided by family doctors: primary dimensions and an attempt at typology
Source: BMC Health Serv Res. 2009 Apr 16;9:63. doi: 10.1186/1472-6963-9-63 (PMC2678111; doi:10.1186/1472-6963-9-63)
Supplement: Additional file 1 — List of positive and negative statements in the respective categories. The list of positive and negative statements in the respective categories is presented. [file 1472-6963-9-63-S1.doc]

Additional file 1

List of positive and negative statements in the respective categories

| Interview number | Personality traits | Competence | Doctor-pat  interaction | Contextual factors | General assessment | Total |
| --- | --- | --- | --- | --- | --- | --- |
| 1. + *ve*  - *ve* | 11  3 | 9  0 | 5  5 | 0  5 | 4  0 | **29**  **13** |
| 2. + *ve*  - *ve* | 4  1 | 0  0 | 16  5 | 0  6 | 5  1 | **25**  **13** |
| 3. + *ve*  - *ve* | 2  2 | 0  0 | 6  8 | 1  0 | 2  1 | **11**  **11** |
| 4. + *ve*  - *ve* | 0  2 | 1  1 | 10  14 | 1  3 | 4  4 | **16**  **24** |
| 5. + *ve*  - *ve* | 5  0 | 1  0 | 11  8 | 5  8 | 3  2 | **25**  **18** |
| 6. + *ve*  - *ve* | 3  0 | 0  0 | 2  4 | 1  5 | 2  1 | **8**  **10** |
| 7. + *ve*  - *ve* | 1  0 | 0  4 | 5  1 | 2  6 | 1  3 | **9**  **14** |
| 8. + *ve*  - *ve* | 3  3 | 5  7 | 16  16 | 3  7 | 9  3 | **36**  **36** |
| 9. + *ve*  - *ve* | 1  1 | 4  4 | 1  10 | 7  7 | 3  1 | **16**  **23** |
| 10. + *ve*  - *ve* | 2  0 | 2  1 | 8  5 | 4  11 | 2  2 | **18**  **19** |
| 11. + *ve*  - *ve* | 5  3 | 3  5 | 10  41 | 3  13 | 0  6 | **21**  **68** |
| 12. + *ve*  - *ve* | 4  0 | 0  4 | 0  27 | 0  2 | 0  0 | **4**  **33** |
| 13. + *ve*  - *ve* | 0  0 | 3  0 | 3  7 | 0  13 | 1  0 | **7**  **20** |
| 14. + *ve*  - *ve* | 0  0 | 0  3 | 1  1 | 1  9 | 2  2 | **4**  **15** |
| 15. + *ve*  - *ve* | 6  0 | 0  14 | 9  8 | 2  5 | 0  0 | **17**  **27** |
| 16. + *ve*  - *ve* | 2  1 | 1  3 | 6  4 | 1  8 | 3  3 | **13**  **19** |
| 17. + *ve*  - *ve* | 1  0 | 4  5 | 4  1 | 2  1 | 6  2 | **17**  **9** |
| 18. + *ve*  - *ve* | 1  1 | 0  1 | 0  5 | 1  10 | 3  2 | **5**  **19** |
| 19. + *ve*  - *ve* | 3  1 | 10  3 | 7  8 | 2  4 | 7  1 | **29**  **17** |
| 20. + *ve*  - *ve* | 0  4 | 6  4 | 15  10 | 5  8 | 7  4 | **33**  **30** |
| 21. + *ve*  - *ve* | 9  0 | 8  2 | 6  8 | 3  0 | 5  0 | **31**  **10** |
| 22. + *ve*  - *ve* | 8  4 | 2  3 | 11  22 | 0  1 | 1  2 | **22**  **32** |

List of positive and negative statements cont

| Interview number | Personality features | Competence | Doctor-pat  interaction | Contextual factors | General assessment | Total |
| --- | --- | --- | --- | --- | --- | --- |
| 23. + *ve*  - *ve* | 2  0 | 3  3 | 4  4 | 0  1 | 6  2 | **15**  **10** |
| 24. + *ve*  - *ve* | 0  0 | 1  0 | 5  5 | 2  4 | 4  2 | **12**  **11** |
| 25. + *ve*  - *ve* | 6  0 | 7  1 | 18  1 | 6  6 | 7  4 | **44**  **12** |
| 26. + *ve*  - *ve* | 2  1 | 0  0 | 12  2 | 9  1 | 7  1 | **30**  **5** |
| 27. + *ve*  - *ve* | 2  0 | 5  4 | 5  3 | 7  7 | 3  0 | **22**  **14** |
| 28. + *ve*  - *ve* | 0  0 | 1  1 | 1  2 | 2  6 | 12  1 | **16**  **10** |
| 29. + *ve*  - *ve* | 2  1 | 1  0 | 17  0 | 8  6 | 10  2 | **38**  **9** |
| 30. + *ve*  - *ve* | 1  4 | 5  4 | 10  2 | 2  3 | 3  2 | **21**  **15** |
| 31. + *ve*  - *ve* | 2  0 | 2  0 | 6  4 | 1  1 | 3  0 | **14**  **5** |
| 32. + *ve*  - *ve* | 0  0 | 0  1 | 0  5 | 0  2 | 1  0 | **1**  **8** |
| 33. + *ve*  - *ve* | 2  0 | 0  0 | 14  1 | 2  1 | 11  1 | **29**  **3** |
| 34. + *ve*  - *ve* | 8  4 | 1  0 | 4  3 | 3  0 | 8  0 | **24**  **7** |
| 35. + *ve*  - *ve* | 2  0 | 2  3 | 5  4 | 1  6 | 4  1 | **14**  **14** |
| 36. + *ve*  - *ve* | 0  1 | 0  0 | 2  5 | 5  7 | 6  0 | **13**  **13** |
| **Positive** | **100** | **87** | **255** | **92** | **155** | **689** |
| **Negative** | **37** | **81** | **259** | **183** | **56** | **616** |
